# A Fatal Case of Anti-PL-7 Antibody-Associated Rapidly Progressive Interstitial Lung Disease Complicated by Tuberculosis: A Case Report

**DOI:** 10.7759/cureus.92421

**Published:** 2025-09-16

**Authors:** Junko Itano, Goro Kimura

**Affiliations:** 1 Department of Allergy and Respiratory Medicine, National Hospital Organization (NHO) Minami-Okayama Medical Center, Okayama, JPN

**Keywords:** anti-pl-7 antibody, immunosuppressive treatment, interstitial lung disease, mycobacterium tuberculosis, rapidly progressive interstitial lung disease

## Abstract

The anti-PL-7 antibody is an anti-aminoacyl-tRNA synthetase antibody. Patients who test positive for anti-PL-7 antibodies present with various clinical symptoms, including myositis, polyarthritis, and interstitial lung disease (ILD). The anti-PL-7 antibody also causes rapidly progressive ILD (RP-ILD), which may be fatal.

In this report, we present the case of a 92-year-old woman with anti-PL-7 antibody-positive RP-ILD, complicated by *Mycobacterium tuberculosis* infection. Treatment for anti-PL-7 antibody-positive RP-ILD typically requires combination therapy with corticosteroids and immunosuppressive agents; however, we were unable to escalate immunosuppression due to concomitant *M. tuberculosis* infection, and the patient died due to respiratory failure. To our knowledge, this is the first report to present the progression of chest computed tomography findings and clinical course of anti-PL-7 antibody-positive RP-ILD complicated by pulmonary tuberculosis. Anti-PL-7 antibody-positive RP-ILD complicated by pulmonary tuberculosis can be fatal. Therefore, clinicians should closely monitor for pulmonary tuberculosis in anti-PL-7 antibody-positive ILD and provide preventive treatment when appropriate.

## Introduction

Idiopathic inflammatory myopathies (IIMs) are autoimmune diseases characterized by inflammation of various organs, including the proximal muscles, skin, joints, and lungs [1,2]. Recently, several myositis-specific antibodies have been identified, including anti-aminoacyl-tRNA synthetase (ARS) antibodies [1,2]. Patients positive for anti-ARS antibodies typically present with various clinical symptoms, including myositis, polyarthritis, and interstitial lung disease (ILD), collectively referred to as antisynthetase syndrome (ASS) [1,2]. Moreover, ILD in ASS patients can develop into rapidly progressive ILD (RP-ILD) [3], which warrants careful monitoring of the clinical course. Anti-ARS antibodies target enzymes involved in protein synthesis, and eight types have been identified: PL-7, Jo-1, PL-12, OJ, EJ, KS, Zo, and Ha [2]. Anti-PL-7 antibodies have been detected in 5%-10% of patients with IIMs [1,2], and 70%-80% of these patients developed ILD [3]. Anti-PL-7 antibody also causes RP-ILD, which requires intensive immunosuppressive treatment [4]. RP-ILD can be fatal, especially in super-elderly patients when complicated by pulmonary tuberculosis. 

This study was approved by the Institutional Ethics Committee of Minami-Okayama Medical Center (2025-28). The patient's family provided written informed consent for publication of this case report.

## Case presentation

A 92-year-old woman presented to a small community hospital with a two-week history of cough and dyspnea. She was diagnosed with acute respiratory failure associated with pneumonia and was admitted to the hospital. Two days after admission, her sputum smear was positive for acid-fast bacilli (AFB), and the polymerase chain reaction (PCR) test for *Mycobacterium tuberculosis* was positive. She was transferred to our hospital for treatment of pulmonary tuberculosis. Her medical history included hypertension, osteoporosis, spondylosis, cataract, and glaucoma. At the age of 12, her chest X-ray showed small nodules, indicating old inflammatory scars, and she was observed without treatment. She had never been diagnosed with ILD and had no recent chest radiographs. She had no history of smoking, pet ownership, or excessive dust exposure, and no family history of pulmonary tuberculosis or ILD.

The patient's temperature was 36.7°C, heart rate 93 beats per minute, blood pressure 104/63 mmHg, and percutaneous arterial oxygen saturation 93%, with nasal oxygen at 2 L/min. Coarse crackles were audible during inhalation. Physical examination revealed no rash, joint pain, or muscle weakness. Table 1 presents the laboratory data.

**Table 1:** Laboratory data Abbreviations: AFB, acid-fast bacilli; Alb, albumin; ALT, alanine aminotransferase; APTT, activated partial thromboplastin time; AST, aspartate aminotransferase; BNP, brain natriuretic peptide; BUN, blood urea nitrogen; CEA, carcinoembryonic antigen; CK, creatine kinase; Cre, creatinine; COVID-19, coronavirus-2019; CRP, C-reactive protein; CYFRA, cytokeratin 19 fragment; FBS, fasting blood sugar; FDP, fibrin/fibrinogen degradation products, HbA1c, hemoglobin A1c; INH, isoniazid; KL-6, Krebs von den Lungen-6; LDH, lactate dehydrogenase; MAC, *Mycobacterium avium *complex; MITOGEN, positive control; % Neutrophils, percentage of neutrophils; Nil, negative control; PCR, polymerase chain reaction; PT, prothrombin time; QFT, QuantiFERON-TB Gold Plus test; RBC, red blood cells; RIF, rifampicin; SP-D, surfactant protein D; TB 1, tuberculosis antigen tube 1; TB 2, tuberculosis antigen tube 2; TP, total protein; WBCs, white blood cells

Blood tests	Results	Reference range
WBC	12.9 × 10^3^/μL	3.3-8.6 × 10^3^/μL
% Neutrophils	98.1%	36.0-69.5%
RBC	445 × 10^4^/μL	386-492 × 10^4^/μL
Hemoglobin	10.9 g/dL	11.6-14.8 g/dL
Platelet	172 × 10^3^/μL	158-348 × 10^3^/μL
TP	6.0 g/dL	6.6-8.1 g/dL
Alb	2.1 g/dL	4.1-5.1 g/dL
AST	32 U/L	13-30 U/L
ALT	19 U/L	7.0-23.0 U/L
CRP	9.70 mg/dL	0.00-0.14 mg/dL
Procalcitonin	6.29 ng/mL	0.00-0.50 ng/mL
LDH	371 U/L	124-222 U/L
BUN	30.0 mg/dL	8.0-20.0 mg/dL
Cre	1.03 mg/dL	0.46-0.79 mg/dL
Ferritin	457 ng/mL	10-160 ng/mL
CK	117 U/L	41-153 U/L
FBS	293 mg/dL	70-140 mg/dL
HbA1c	6.3%	4.9-6.0%
BNP	79.0 pg/mL	0.0-18.4 pg/mL
KL-6	2972 U/mL	0-500 U/mL
SP-D	862 ng/mL	0-109 ng/mL
CEA	12.1 ng/mL	0.0-5.0 ng/mL
CYFRA	10.2 ng/mL	0.0-3.5 ng/mL
PT	13.8 sec	10.0-14.4 sec
APTT	33.8 sec	24.0-40.0 sec
Fibrinogen	302.8 mg/dL	150.0-400.0 mg/dL
FDP	63.6 μg/mL	0.0-5.0 μg/mL
D-dimer	30.3 μg/mL	0.0-1.0 μg/mL
(1→3)-β-D-glucan	13.9 pg/mL	0.0-20.0 pg/mL
Anti-MAC antibody	(-)	(-)
*Aspergillus* antigen	(-)	(-)
*Cryptococcus* antigen	(-)	(-)
QFT	indeterminate	(-)
MITOGEN	< 0.05 IU/mL	-
TB 1	< 0.05 IU/mL	0.00-0.35 IU/mL
TB 2	< 0.05 IU/mL	0.00-0.35 IU/mL
Nil	< 0.05 IU/mL	-
Others	Results	Reference range
Nasal swab tests		
COVID-19 antigen	(-)	(-)
Influenza antigen	(-)	(-)
Urinary antigen tests	(-)	(-)
Streptococcus pneumoniae	(-)	(-)
Legionella	(-)	(-)
Blood culture test	(-)	(-)
Sputum tests		
Bacterial culture	Escherichia coli	(-)
AFB	Gaffky 5	(-)
TB-PCR	(+)	(-)
MAC-PCR	(-)	(-)
RIF/INH resistance assay	(-)	(-)
Cytology	(-)	(-)

There was an increase in white blood cells (WBCs) with a predominance of neutrophils, C-reactive protein (CRP), and procalcitonin values, indicating a pronounced inflammatory response. Furthermore, markers of ILD - such as lactate dehydrogenase (LDH), Krebs von den Lungen-6, and surfactant protein D - also increased. Fibrin/fibrinogen degradation products and D-dimer levels were elevated, and there was also the presence of thrombi in both the femoral and saphenous veins. Carcinoembryonic antigen and cytokeratin 19 fragment levels increased, while sputum cytology was negative. Serum anti-*Mycobacterium avium *complex (MAC) antibody, *Aspergillus* antigen, and *Cryptococcus* antigen tests were negative. The interferon-gamma (IFN-γ) release assay using the QuantiFERON-TB Gold Plus test indicated indeterminate results. Nasal swab antigen tests for coronavirus-2019 and influenza virus were negative, as were urinary antigen tests for *Streptococcus* *pneumoniae* and *Legionella*. Blood culture results were negative. *Escherichia coli *was detected in sputum cultures. The sputum smear showed AFB (Gaffky 5), and PCR for *M. tuberculosis* was positive, while PCR for MAC was negative. Real-time PCR testing using cobas® MTB-RIF/INH reagents revealed no resistance gene mutations for isoniazid (INH) and rifampicin (RIF).

**Figure 1:**
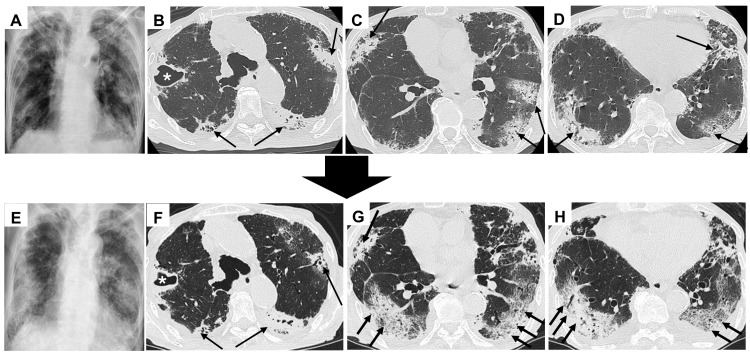
Chest images obtained from the previous hospital and our hospital (A-D) Chest images from the previous hospital. (A) Chest X-ray showing consolidations and ground-glass opacities (GGOs) in both lung fields. (B-D) High-resolution computed tomography (HRCT) images. (B) HRCT shows a cavity and granular opacities in the right upper lobe (asterisk), along with consolidation and GGOs in the bilateral upper lobes (arrows). HRCT also shows patchy consolidations and GGOs, predominantly in the lower lung peripheries (arrows). (E-H) Chest images from our hospital obtained two days after the previous imaging. (E) Chest X-ray showing extensive consolidation and GGOs in both lung fields. (F) HRCT shows a cavity and granular opacities in the right upper lobe (asterisk), consolidation, and GGOs in the bilateral upper lobes (arrows). HRCT images show more extensive consolidation and GGOs than the previous images, predominantly in patchy areas around the lower lobes of both lungs (arrows).

Chest imaging findings are shown in Figure 1, with data from the previous hospital (Figures 1A-1D) and our hospital (Figures 1E-1H), obtained at a two-day interval. Chest radiography revealed consolidation in both lung fields (Figures 1A, 1E). Chest high-resolution computed tomography (HRCT) showed cavities and granular opacities in the right upper lobe (Figures 1B, 1F), indicating *M. tuberculosis* infection. HRCT images also demonstrated patchy consolidations and ground-glass opacities (GGOs) in the lower lung peripheries, suggesting ILD with an organizing pneumonia (OP) pattern (Figures 1B-1D). Two days later, the GGOs and consolidations in the lower lung peripheries had rapidly expanded, indicating acute exacerbation of ILD with an OP pattern (Figures 1F-1H).

**Figure 2:**
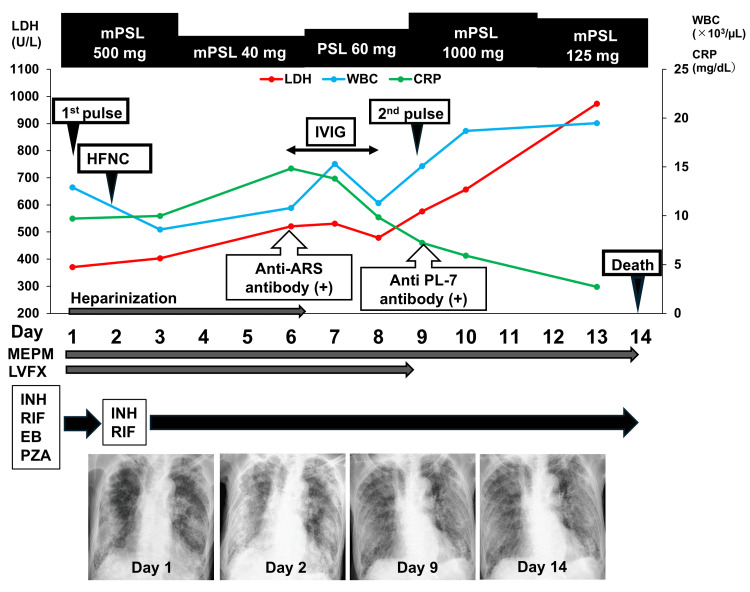
Patient's treatment course, radiologic, and laboratory information Abbreviations: ARS, aminoacyl-tRNA synthetase; CRP, C-reactive protein; EB, ethambutol; HFNC, high-flow nasal cannula; INH, isoniazid; IVIG, intravenous immunoglobulin; LDH, lactate dehydrogenase; LVFX, levofloxacin; MEPM, meropenem; mPSL, methylprednisolone; Pulse, corticosteroid pulse therapy; PZA, pyrazinamide; RIF, rifampicin; WBCs, white blood cells

The diagnosis was respiratory failure due to exacerbation of pulmonary tuberculosis and acute exacerbation of ILD with an OP pattern. The patient's treatment course, radiologic, and laboratory information are shown in Figure 2.

Anti-tuberculosis treatment included INH, RIF, ethambutol (EB), and pyrazinamide (PZA). Given the severe bacterial pneumonia, including *E. coli* detected in her sputum, we initiated antibiotic therapy with meropenem and levofloxacin. Corticosteroid pulse therapy with 500 mg of methylprednisolone (mPSL) was started for the acute exacerbation of ILD at the previous hospital for two days and was continued at the same dose after the patient was transferred. Thrombolytic therapy with heparin was initiated due to thrombosis in both femoral and saphenous veins associated with severe infection.

On the day following admission, the patient’s respiratory failure worsened significantly, requiring 100% fraction of inspired oxygen (FiO_2_) via high-flow nasal cannula (HFNC). She developed a persistent high fever, elevated WBC count, and CRP levels; LDH levels also increased beyond admission values. Renal function worsened, necessitating discontinuation of EB and PZA on day 3, while INH and RIF were continued. On day 6, the autoimmune antibody screening test revealed a positive result for the anti-ARS antibody (index 39.3) by enzyme-linked immunosorbent assay. In contrast, all other autoimmune antibodies were negative, as shown in Table 2.

**Table 2:** Results of the autoimmune antibody test Abbreviations: ANA, antinuclear antibody; ARS, aminoacyl-tRNA synthetase; CCP, cyclic citrullinated peptide; dsDNA, double-stranded DNA; GBM, glomerular basement membrane; MDA5, melanoma differentiation-associated gene 5; MPO-ANCA, myeloperoxidase-antineutrophil cytoplasmic antibody; PR3-ANCA, proteinase 3-antineutrophil cytoplasmic antibody; RNP, ribonucleoprotein; Scl-70, topoisomerase I; SRP, signal recognition particle; SS, Sjögren’s syndrome; TIF-γ, transcription intermediary factor 1-gamma

Autoimmune antibody	Results
ANA	Titer < 1:40
Anti-SS-A/Ro antibody	(-)
Anti-SS-B/La antibody	(-)
Anti-Sm antibody	(-)
Anti-dsDNA antibody	(-)
Anti–Scl-70 antibody	(-)
Anti–RNP antibody	(-)
Anti-CCP antibody	(-)
MPO-ANCA	(-)
PR3-ANCA	(-)
Anti-GBM antibody	(-)
Anti-cardiolipin antibody	(-)
Anti-ARS antibody	Index 39.3
Anti-MDA5 antibody	(-)
Anti-TIF-γ antibody	(-)
Immunoblotting method	
Anti-Mi-2 antibody	(-)
Anti-Ku antibody	(-)
Anti-PM-Scl100 antibody	(-)
Anti-PM-Scl75 antibody	(-)
Anti-SRP antibody	(-)
Anti-Jo-1 antibody	(-)
Anti-PL-7 antibody	(+)
Anti-PL-12 antibody	(-)
Anti-OJ antibody	(-)
Anti-EJ antibody	(-)
Anti-Ro-52 antibody	(-)

On day 9, immunoblotting revealed that the anti-PL-7 antibody was positive, while all other autoantibodies were negative, as shown in Table 2. The patient was diagnosed with anti-PL-7 antibody-positive RP-ILD. After the first corticosteroid pulse therapy, the corticosteroid dose was reduced to 40 mg of mPSL. Following the positive test result for anti-ARS antibodies, the prednisolone dose was increased to 60 mg. Furthermore, after confirmation of anti-PL-7 antibody positivity, a second corticosteroid pulse therapy with 1000 mg of mPSL was initiated, and the dose was then maintained at 125 mg of mPSL. Her respiratory failure did not improve, and her chest radiograph showed progression of GGO and consolidation, as shown in Figure 2.

Due to complications from *M. tuberculosis* and *E. coli* infections, we were unable to intensify immunosuppressive treatment for anti-PL-7 antibody-positive RP-ILD. In addition to corticosteroids, intravenous immunoglobulin, consisting of freeze-dried immunoglobulin G, was administered at a dose of 2,500 mg from days 6 to 8. The patient and her family declined intubation and mechanical ventilation. Her respiratory status continued to be supported with 100% FiO_2_ via HFNC; however, the patient died 14 days after admission. Approximately two months after her death, drug susceptibility test results for the sputum strain were obtained using Bit Spectre-SR (Kyokuto Pharmaceutical Industrial, Tokyo, Japan) and PZA broth (Kyokuto Pharmaceutical Industrial). The sputum colony was resistant to EB at 2.5 μg/mL but sensitive to all other anti-tuberculosis drugs, including INH, RIF, and PZA.

## Discussion

Anti-PL-7 antibody is an anti-ARS antibody targeting threonyl-tRNA synthetase and is associated with various clinical symptoms, including myositis and ILD, as defined by ASS [1,2]. Approximately 70% of ASS patients with anti-PL-7 antibodies present with ILD [3]. In our case, no skin rash or muscle weakness suggestive of polymyositis/dermatomyositis (PM/DM) was observed, and the clinical symptoms were limited to ILD. A previous report found that 35.8% of ILD patients who tested positive for anti-PL-7 antibody only had ILD and no symptoms of PM/DM [5]. Radiological patterns of patients with ILD most frequently include nonspecific interstitial pneumonia (NSIP), followed by OP [6,7]. ILD lesions with the NSIP pattern in ASS are typically located in the lower lobes, periphery, and/or peribronchovascular regions. ILD lesions with the OP pattern in ASS typically show patchy areas of peripheral, subpleural, and peribronchiolar consolidations [6]. In our case, HRCT revealed that consolidations and GGOs were predominantly in patchy areas of both lower lung peripheries, corresponding to an OP pattern of ILD.

Typical HRCT findings in patients with pulmonary tuberculosis include granular opacities, thick-walled cavities, and centrilobular nodules [8]. However, previous reports have occasionally described an OP pattern on HRCT in patients with pulmonary tuberculosis [8,9]. The OP pattern in such cases is rare and typically presents as localized consolidation and GGO lesions in the lungs [9-11]. It often appears as single or multiple reversed halo signs with ring-band consolidation, predominantly located in the upper and middle lobes of the lung [11]. In our patient, GGOs and consolidations, indicating an OP pattern, were detected and had expanded in the dominant lower lung peripheries. Moreover, according to a previous report, sputum smears and bronchoalveolar lavage fluid samples in patients with pulmonary tuberculosis and an OP pattern on HRCT often yield negative results [11,12]. In contrast, our patient’s sputum smears tested positive for AFB (Gaffky 5). Based on the above, the results of these HRCT findings and sputum tests were all atypical for pulmonary tuberculosis with an OP pattern. Therefore, we believe that the fatal respiratory failure was caused by active pulmonary tuberculosis and anti-PL-7 antibody-positive ILD.

Anti-PL-7 antibody-positive ILD is often associated with RP-ILD, which is characterized by rapid clinical deterioration, progressive HRCT abnormalities, and respiratory failure [3,4]. Our patient had anti-PL-7 antibody-positive ILD with an OP pattern on HRCT and was diagnosed with RP-ILD based on the rapid worsening of HRCT findings and respiratory failure. Treatment for RP-ILD typically requires combination therapy, including corticosteroids and immunosuppressive agents such as tacrolimus, azathioprine, mycophenolate mofetil, rituximab, cyclophosphamide, calcineurin inhibitors, and Janus kinase inhibitors [3,4,13]. However, patients receiving these immunosuppressants are at high risk of reactivating latent *M. tuberculosis* infection [14,15], and tuberculosis preventive treatment (TPT) for latent tuberculosis infection (LTBI) should be administered before initiating immunosuppressive therapy [16]. 

Based on our PubMed search, only one reported case of anti-PL-7 antibody-positive ILD with concurrent pulmonary tuberculosis has been reported [17]; however, the clinical HRCT findings and clinical course in that case were unknown. Aside from our report, to our knowledge, no studies have described the incidence or clinical course of pulmonary tuberculosis in patients with anti-PL-7 antibody-positive ASS, and the characteristics of such cases remain unclear.

There is no established evidence for the treatment of pulmonary tuberculosis complicated with RP-ILD due to the lack of clinical experience. In general, we should avoid administering immunosuppressants during treatment for pulmonary tuberculosis because they can cause fatal inflammatory reactions. Ho and Leung [18] reported on the treatment of pulmonary tuberculosis coexisting with lung cancer. They recommend that patients with early- or latent-stage lung cancer undergo a two- to three-week course of pretreatment with anti-tuberculosis drugs before lung cancer treatment [18]. In this case, the patient had a severe *E. coli *infection and worsening renal dysfunction, preventing standard tuberculosis treatment. The patient died from severe respiratory failure.

As demonstrated in our case, patients with anti-PL-7 antibody-positive ILD and complications such as pulmonary tuberculosis can be fatal. Therefore, in patients with anti-PL-7 antibody-positive ILD, clinicians should remain vigilant for such infections. Furthermore, a thorough medical history, including prior *M. tuberculosis* infection and family history, should be obtained. In addition to follow-up chest X-rays or HRCT, screening tests such as the IFN-γ release assay should be performed in anti-PL-7 antibody-positive ILD patients. TPT should be considered in those at high risk of reactivating latent *M. tuberculosis* infection.

## Conclusions

In this report, we presented a case of anti-PL-7 antibody-positive RP-ILD complicated by pulmonary tuberculosis. The co-morbidity rate of anti-PL-7 antibody-positive RP-ILD and pulmonary tuberculosis remains unclear, and to our knowledge, this is the first report to describe the clinical HRCT findings and clinical course of this complication. Anti-PL-7 antibody-positive RP-ILD complicated by pulmonary tuberculosis can be fatal if we cannot intensify immunosuppression for the treatment of RP-ILD. Therefore, clinicians should be aware of the risk of complications from *M. tuberculosis* infections and regularly perform chest X-rays, sputum AFB tests, and IFN-γ release assays on anti-PL-7 antibody-positive ILD patients undergoing intense immunosuppressive treatment. Furthermore, TPT should be considered for patients with LTBI to prevent the onset of pulmonary tuberculosis.

## References

[1] Lundberg IE, Fujimoto M, Vencovsky J (2021). Idiopathic inflammatory myopathies. Nat Rev Dis Primers.

[2] Wu Y, Luo J, Duan L (2024). Pathogenic mechanisms of disease in idiopathic inflammatory myopathies: autoantibodies as clues. Front Immunol.

[3] Moda M, Yanagihara T, Nakashima R, Sumikawa H, Shimizu S, Arai T, Inoue Y (2025). Idiopathic inflammatory myopathies-associated interstitial lung disease in adults. Tuberc Respir Dis (Seoul).

[4] Yanagihara T, Suzuki K, Egashira A (2020). Nintedanib and intensive immunosuppressive therapy to treat rapidly progressive interstitial lung disease presenting anti-ARS antibodies. Respir Med Case Rep.

[5] Marie I, Josse S, Decaux O (2013). Clinical manifestations and outcome of anti-PL7 positive patients with antisynthetase syndrome. Eur J Intern Med.

[6] Waseda Y, Johkoh T, Egashira R (2016). Antisynthetase syndrome: pulmonary computed tomography findings of adult patients with antibodies to aminoacyl-tRNA synthetases. Eur J Radiol.

[7] Patel P, Marinock JM, Ajmeri A, Brent LH (2024). A review of antisynthetase syndrome-associated interstitial lung disease. Int J Mol Sci.

[8] Wetscherek MT, Sadler TJ, Lee JY, Karia S, Babar JL (2022). Active pulmonary tuberculosis: something old, something new, something borrowed, something blue. Insights Imaging.

[9] Zhao X, Cheng Y, Xiong Y, Wang G (2023). Pulmonary tuberculosis associated acute fibrinous and organizing pneumonia: a case report and literature review. Clin Respir J.

[10] Huang LL, Wang C, Liu Y, Gu XY, Wang WX, Chen W, Hu CM (2023). Resolution of an insidious and migratory Mycobacterium tuberculosis-associated secondary organizing pneumonia: a case report and literature review. BMC Infect Dis.

[11] Zeng Y, Zhai XL, Wáng YX (2021). Illustration of a number of atypical computed tomography manifestations of active pulmonary tuberculosis. Quant Imaging Med Surg.

[12] Kim EJ, Kim KC (2020). Pulmonary tuberculosis presenting secondary organizing pneumonia with organized polypoid granulation tissue: case series and review of the literature. BMC Pulm Med.

[13] Thong L, Chawke LJ, Murphy G, Henry MT (2023). Management of myositis associated interstitial lung disease. Rheumatol Int.

[14] Bhattacharya PK, Jamil M, Roy A, Talukdar KK (2017). SLE and tuberculosis: a case series and review of literature. J Clin Diagn Res.

[15] Chiu YM, Chen DY (2020). Infection risk in patients undergoing treatment for inflammatory arthritis: non-biologics versus biologics. Expert Rev Clin Immunol.

[16] Fehily SR, Al-Ani AH, Abdelmalak J (2022). Review article: latent tuberculosis in patients with inflammatory bowel diseases receiving immunosuppression-risks, screening, diagnosis and management. Aliment Pharmacol Ther.

[17] Sato S, Hirakata M, Kuwana M (2005). Clinical characteristics of Japanese patients with anti-PL-7 (anti-threonyl-tRNA synthetase) autoantibodies. Clin Exp Rheumatol.

[18] Ho JC, Leung CC (2018). Management of co-existent tuberculosis and lung cancer. Lung Cancer.

