# Correction: A Fatal Case of Anti-PL-7 Antibody-Associated Rapidly Progressive Interstitial Lung Disease Complicated by Tuberculosis: A Case Report

**DOI:** 10.7759/cureus.c431

**Published:** 2026-05-08

**Authors:** Junko Itano, Goro Kimura

**Affiliations:** 1 Department of Allergy and Respiratory Medicine, National Hospital Organization (NHO) Minami-Okayama Medical Center, Okayama, JPN

This article has been corrected to change the authors’ affiliation to the Department of Allergy and Respiratory Medicine, National Hospital Organization Minami-Okayama Medical Center, Okayama, JPN.

